# A Comparative Study on Genotoxic and Oxidative DNA Damage in Oral Epithelial Cells of COVID-19 Suspected Patients

**DOI:** 10.7759/cureus.82593

**Published:** 2025-04-19

**Authors:** Yogitha Poojari, Senthil Murugan, P K Sankaran, Joy A Ghoshal, Yuvaraj Maria Francis, Mohan K A, Vinoth K Kalidoss

**Affiliations:** 1 Anatomy, All India Institute Of Medical Sciences, Mangalagiri, IND; 2 Anatomy, All India Institute of Medical Sciences, Mangalagiri, IND; 3 Anatomy, Saveetha Institute of Medical and Technical Sciences, Chennai, IND; 4 Medical Microbiology, All India Institute Of Medical Sciences, Mangalagiri, IND; 5 Community and Family Medicine, All India Institute of Medical Sciences, Mangalagiri, IND

**Keywords:** 8 hydroxy deoxy guanosine, buccal cells, covid-19, oxidative dna damage, sars-cov2

## Abstract

Introduction

The SARS-CoV-2 virus causes COVID-19 by chiefly infecting the nasal and oral mucosal cavities, where its spike proteins bind to angiotensin-converting enzyme II (ACE2) receptors on epithelial cells. This study aimed to assess SARS-CoV-2-related genotoxicity in buccal mucosal cells through micronuclei counts and 8-Oxo-2'-deoxyguanosine (8-OHdG) expression.

Methods

This cross-sectional study was conducted in 86 COVID-19 suspected patients aged 18-45 years attending an outpatient screening area for reverse transcription polymerase chain reaction (RT-PCR) SARS-CoV-2 testing between December 2023 and February 2024. Salivary swabs were obtained and smeared on glass slides for each patient. These slides were stained to study and compare cellular toxicity by Papanicolaou (Pap) staining and oxidative DNA damage expression by immunohistochemistry. The categorical and continuous variables were assessed using the independent t-test and ANOVA. A p-value of less than 0.05 was considered statistically significant.

Results

The number of micronucleated cells, total micronuclei, neutrophil, lymphocyte and inflammatory cell count in COVID-19 RT-PCR positive patients were 11.32 ± 7.00, 17.32± 12.53, 1.93 ± 1.17, 3.98 ± 2.55 and 5.90 ± 3.15 respectively which is higher than COVID-19 RT-PCR negative individuals 6.09± 3.83, 9.04± 5.82, 0.18± 0.49, 2.13± 0.84 and 2.31±1.01 respectively. Moreover, buccal cells showed increased oxidative DNA damage with intense staining for 8-OHdG in COVID-19-positive patients. The epithelial-to-inflammatory cell ratio was very low in COVID-19-positive buccal smear patients.

Conclusion

This study concludes that SARS-CoV-2 has a genotoxic effect by increasing the micronuclei count and has oxidative DNA damage by increasing the 8-OHdG expression in the exfoliated buccal cells.

## Introduction

The advent of the novel coronavirus disease 2019 (COVID-19), instigated by the severe acute respiratory syndrome coronavirus 2 (SARS-CoV-2), exhibits a global health crisis of extraordinary proportions, challenging healthcare systems worldwide and profoundly impacting human health and well-being. As of February 2024, more than 774 million confirmed cases and 7 million mortalities have been reported worldwide [1]. SARS-CoV-2 is an airborne pathogen with spike proteins that bind exclusively to host receptors, such as angiotensin-converting enzyme II receptors (ACE 2) and transmembrane serine protease (TMPRSS) family members existent in the respiratory tract, gastrointestinal tract, myocardium, urinary bladder, and even in the oral mucosa, leading to fever, dry cough, fatigue, dyspnea, sore throat, loss of taste, oral lesions, diarrhea, vomiting, progressive alveolar damage, and death [2]. The virus has been detected in the saliva of patients by reverse transcriptase‐polymerase chain reaction (RT‐PCR) and is considered to be more sensitive than the nasopharyngeal test [3]. Oral epithelial cells, the primary cellular component of the oral mucosa, play a critical role in the host defense against microbial invasion and serve as an integral part of the immune response. Respiratory viral infections, more so with COVID-19, cause genomic instability by releasing excessive cytokines with upsurged levels of inflammation, redox, and immune response instability. Increased inflammation and oxidative stress have aggregate antiviral effects and can cause tissue, oxidative, and DNA damage [4, 5].

Micronuclei

As a result of DNA damage, micronuclei are formed, which are microscopically visible, small, round or oval extranuclear bodies that contain fragments of chromosomes and/or whole chromosomes, and are formed during cell division. The micronucleus assay is a widely used method for assessing genotoxicity and chromosomal instability in cells exposed to various factors such as smoking, tobacco chewing, and alcoholism to identify individuals at a high risk of oral lesions. Changes in micronuclear count can serve as a biomarker of DNA damage and chromosomal instability, reflecting the impact of various environmental and endogenous factors, including viral infections, such as COVID-19 [6].

8-Oxo-2'-deoxyguanosine (8-OHdG)

Oxidative stress can be defined as an imbalance between the production of reactive oxygen species (ROS), such as superoxide anions, hydrogen peroxide, hydroxyl radicals, and singlet oxygen, and depletion of endogenous mechanisms (the antioxidant system). Biomarkers that can imitate the severity of oxidative stress in COVID-19 are advanced oxidation protein products (AOPP) and 8-Oxo-2'-deoxyguanosine (8-OHdG), which are used in the study. 8-OHdG is formed by the oxidation of deoxyguanosine residues within DNA, resulting in the generation of a modified nucleoside that can be detected and quantified using various analytical techniques such as enzyme-linked immunosorbent assay (ELISA) or chromatography coupled with mass spectrometry. It can cross the cell membrane and can be detected in the urine, saliva, and serum. 8-OHdG serves as a sensitive and specific marker for evaluating endogenous oxidative DNA damage that leads to mutagenesis and carcinogenesis, and its concentration may be related to the exacerbation and severity of COVID-19 [7]. Various studies have explored the micronucleus count in diverse environmental toxin exposures, but there are no specific studies correlating the inflammatory response, toxicity, and expression levels of oxidative DNA damage due to SARS-CoV-2. Since the COVID-19 pandemic began in 2019 and is still ongoing, there is a compelling need to comprehend its meticulous pathogenesis. Hence, the present study aimed to gain insights into genotoxicity by measuring the micronuclei count and oxidative DNA damage levels by measuring the expression of 8-OHdG caused by SARS-CoV-2.

## Materials and methods

This cross-sectional research was executed in COVID-19 suspected patients aged 18-45 years attending an outpatient screening area for reverse transcription polymerase chain reaction (RT-PCR) SARS-CoV-2 testing between December 2023 and February 2024 after obtaining institutional ethical clearance (AIIMS/MG/IEC/2023-24/57). A total of 86 COVID-19 suspected patients were included in this study. Asymptomatic patients, those with oral lesions, smokers, alcoholics, and tobacco chewers were excluded. A previous study done for micronuclei count among the control Indian population showed that the mean (SD) micronucleated cells in buccal mucosa cells of COVID-19 patients and controls are 1.4(1.5) and 0.2(0.5). The sample size was calculated to be 41 in each group (expecting similar results in COVID 19 positive patients and expecting a minimum of 50% less mean (0.7) micronucleated cells among controls with 5% alpha error and 80% power) using OpenEpi Version 3.01 Software Software (Bill and Melinda Gates Foundation, United States, 2013). Based on RT-PCR test results, the patients were divided into COVID-19 positive (n=41) and COVID-19 negative (n=45). The buccal smears were obtained using a wooden spatula, scratched gently on the sides of the oral cavity, and smeared on premarked slides. For each patient, two slides were made to study the micronuclei count and oxidative damage expression levels. 

Genotoxic assessment

The buccal scratched and smeared slides were stained with rapid Papanicolaou (Pap) to assess the genotoxicity expressed as micronuclei and other inflammatory cells. The small extracellular nuclear material with the same staining intensity as the nucleus was taken as the micronuclei count, and for counting the micronuclei, a 100x objective was used. As the slides were stained with Pap stain, the epithelial cells were clearly visualized, and only cells with clear margins and nuclei were taken for counting, and the overlapping cells were eliminated. The inflammatory cells out of 100 cells were counted and tabulated for neutrophils, lymphocytes, and other inflammatory cells. The epithelial to inflammatory cell ratio was calculated as follows: epithelial to inflammatory cell ratio = number of normal salivary epithelial cells/ number of Inflammatory cells.

Oxidative DNA damage

The smeared slides were incubated with 2% normal goat serum to prevent background staining and further incubated with an antibody against 8-OHdG after serial dilution ratios. On the next day, the secondary antibody was added after washing with further incubation for 2 hours. With THE ABC reagent and DAB incubation, the staining can be localized and visualized under a microscope. The immunohistochemical expressions of 8-Oxo-2'-deoxyguanosine (8-OHdG; Novus) at a 1:400 dilution ratio within the buccal cells were studied for their intensity and location. The intensity of immunostaining was compared as mild, moderate, and severe using H score [8] using percentage of particular stained cells and between the nucleus and micronucleus, and between the RT-PCR COVID-19 positive and negative patients.

Correlation between cytogenetic and oxidative DNA damage

The micronucleated cell count, other abnormal cell counts, and epithelial to inflammatory cell ratio were compared between COVID-19-positive and -negative patients. The pattern of expression of oxidative DNA damage and its intensity were compared with the micronucleated cell count and epithelial to inflammatory cell ratio.

Statistical analysis

The continuous variables like age, micronucleated cells, and epithelial to inflammatory cell ratio were summarized as mean and standard deviation or median and interquartile range. The categorical variables, like the study groups and gender, were summarized as frequency and proportion. Subgroup analysis between categorical and continuous variables was assessed using the independent t-test and ANOVA. Subgroup analysis between categorical variables was done using the Chi-squared test. The p-values less than 0.05 were considered statistically significant. The data were entered in Excel (Microsoft, Redmond, Washington), and the data were analyzed using SPSS software version 26 (IBM Inc., Armonk, New York).

## Results

Micronucleus and inflammatory cell count

**Figure 1:**
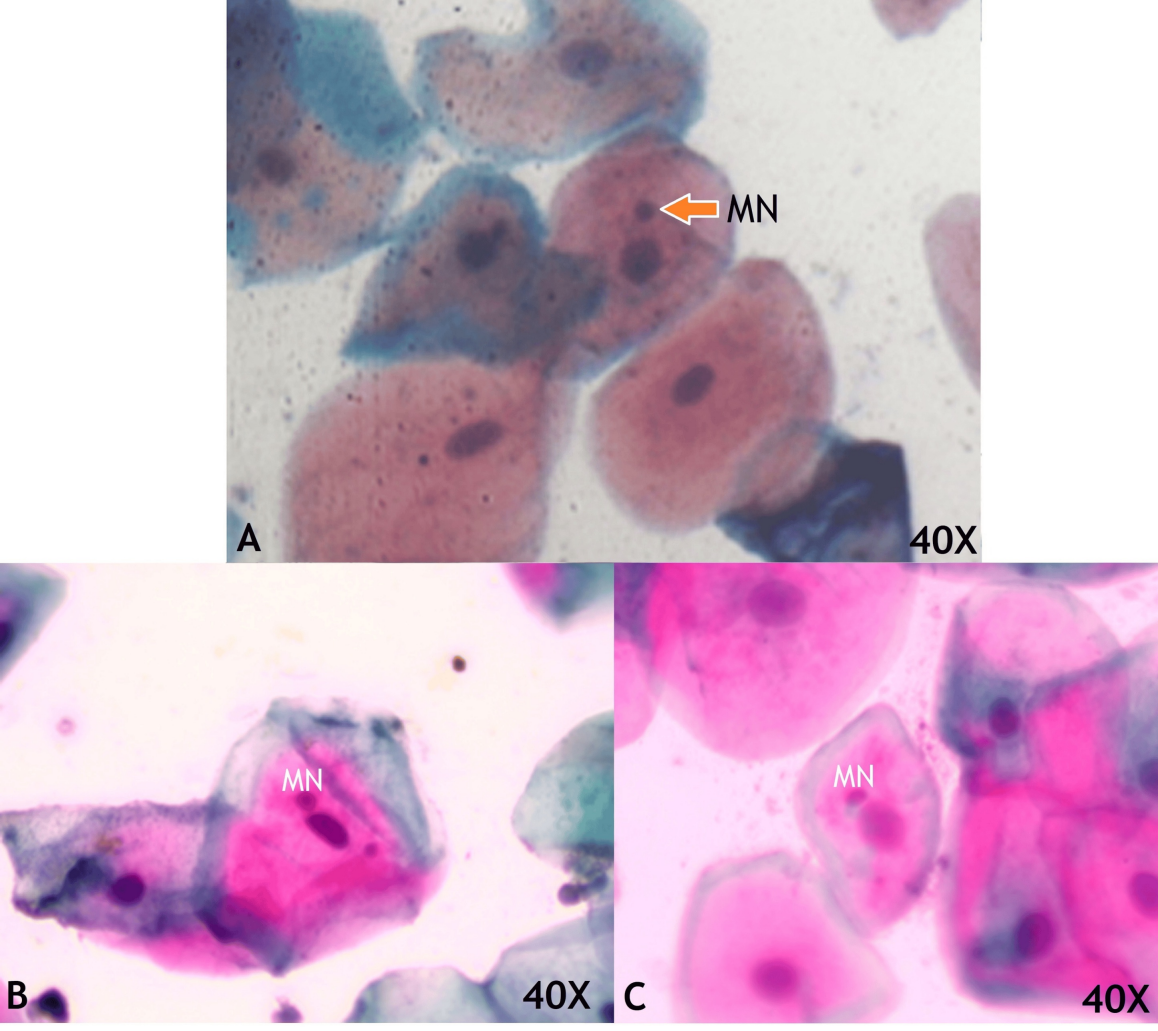
Exfoliated buccal mucosal cells in medium magnification consisting of micronuclei (MN) which are small rounded smooth periphery and same staining intensity of nuclei in COVID-19 negative patients

The exfoliated buccal cells stained with Pap smear showed Eosin-stained cells indicating superficial squamous cells, orangish-stained cells are keratinized squamous cells, and hematoxylin-stained cells are intermediate squamous cells. The few buccal cells revealed additional nuclear material with a rounded appearance, smooth in the periphery, and similar staining intensity to the nucleus, which are micronuclei (MN; Figure 1) in both COVID-19 positive and negative patients.

The number of micronucleated cells and the number of micronuclei per 100 cells in the single smear were counted and tabulated (Table 1).

**Table 1:** The total micronucleated cell count, micronuclei count and inflammatory cell count. The micronucleated cell count and inflammatory cell count are significantly higher in COVID 19 positive patients. p-value of less than 0.05 is significant

Parameter	COVID-report	N	Mean	Std. deviation	p-value (Student t test)
Age	Positive	41	33.46	10.22	0.415
	Negative	45	31.80	8.60	
Micronucleated cell count	Positive	41	11.32	7.00	<0.001
	Negative	45	6.09	3.83	
Total Micronuclei Count	Positive	41	17.32	12.53	<0.001
	Negative	45	9.04	5.82	
Neutrophil count	Positive	41	1.93	1.17	<0.001
	Negative	45	0.18	0.49	
Lymphocyte count	Positive	41	3.98	2.55	<0.001
	Negative	45	2.13	0.84	
Inflammatory cell count	Positive	41	5.90	3.15	<0.001
	Negative	45	2.31	1.01	
Normal cell count	Positive	41	82.78	9.19	<0.001
	Negative	45	91.60	4.10	

**Figure 2:**
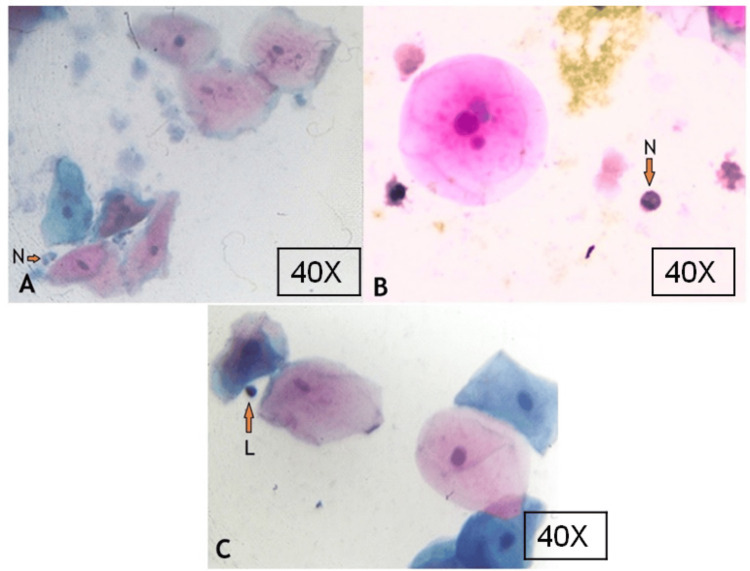
Buccal smear showing buccal cells and inflammatory cells in COVID-19-positive patients N - neutrophils; L - lymphocyte

The average total micronucleated cell count in COVID-19 positive patients was 11.32 ± 7.00, and in COVID-19 negative patients was 6.09 ± 3.83, which was significantly higher in positive patients compared with COVID-19 negative individuals. Apart from flat buccal cells, there are inflammatory cells in the buccal smear of a COVID-19 positive patient (Figure 2) were also counted, and the normal epithelial to inflammatory cell ratio was calculated.

Genotoxic assessment

The epithelial to inflammatory cell ratio was calculated as follows. Epithelial to inflammatory cell ratio = number of normal salivary epithelial cells/number of inflammatory cells. Epithelial to inflammatory cell ratio COVID-19-positive = 82.78/5.90 = 14.03. Epithelial to inflammatory cell ratio for COVID-19 negative = 91.60/2.31 = 39.65. The epithelial to inflammatory cell ratio was very low in COVID-19-positive buccal smear patients.

Oxidative DNA damage

**Figure 3:**
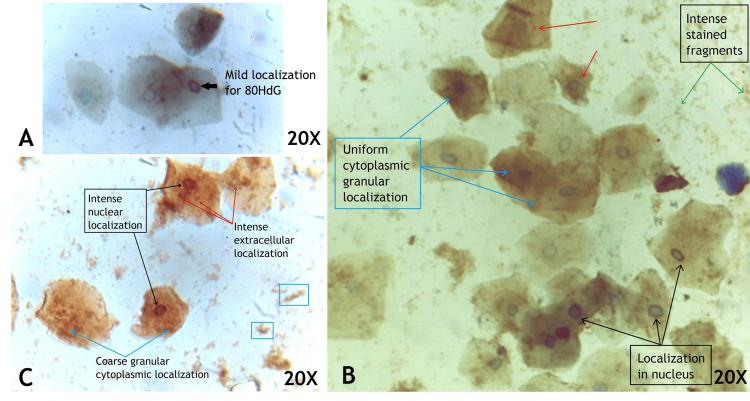
Buccal cells of COVID-19-positive patient localized for 8-OHdG and counter stained with hematoxylin showing mild (a), moderate (b) and intense (c) staining

Immunohistochemical staining for 8 hydroxy deoxy Guanosine (8-OHdG) within the buccal cells was studied for distribution and staining intensity. The buccal smear of COVID-19 negative patients showed mild localization or no localization within the cell cytoplasm or nucleus. In contrast, the buccal cells in COVID-19 positive patients revealed varying intensity from mild (Figure 3a), moderate (Figure 3b) to intense (Figure 3c) [8]. Also, there was fine granular cytoplasmic localization for 8-OHdG and stained fragments outside the cells (Figure 3b). Eleven COVID-19 positive patients showed an intense staining pattern with coarse granules in the cytoplasm and intense staining in the entire nucleus Figure 3c and Table 2.

**Table 2:** Distributes the number of patients based on intensity of staining of 8-OHdG in COVID-19-positive and negative patients 8-OHdG - 8-Oxo-2'-deoxyguanosine

Severity	COVID-positive	COVID-negative (frequency)	Total (frequency)	p-value ANOVA (<0.05 is significant)
Mild	13 (31.7)	44 (97.8)	57 (66.3)	<0.001
Moderate	17 (41.4)	1 (2.2)	18 (21.0)	
Severe	11 (26.8)	0 (0)	11 (12.8)	
Total	41 (47.7)	45 (52.3)	86 (100)	

## Discussion

The COVID-19 pandemic caused by the novel coronavirus SARS-CoV-2 has raised significant concerns regarding its potential impact on oral health, including the possibility of genotoxic and oxidative DNA damage in oral epithelial cells [9]. The accumulation of genotoxic and oxidative DNA damage in oral epithelial cells of COVID-19 suspected patients may have important implications for disease severity and progression [10]. Understanding the molecular mechanisms underlying DNA damage in the oral cavity of COVID-19 suspected patients is crucial for elucidating disease pathogenesis and identifying potential biomarkers of disease severity and progression. The findings of this study revealed remarkable changes in the number of micronucleated, pyknotic, karyolytic, and karyorrhexis cells and expression of 8-OHdG between COVID-19 RT-PCR positive patients and negative patients.

Micronuclei count

The SARS-CoV-2 virus commonly transmits via salivary and respiratory droplets. Additionally, it can also be transmitted through blood, urine, feces, and tears [11]. The access of SARS-CoV-2 into the body by binding with ACE 2 and TMPRSS2 receptors, and they are noticed in the oral mucosa, tongue, and salivary gland [12, 13]. The findings of this study revealed a remarkable increase in the quantity of micronuclei in buccal cells of COVID-19-positive patients compared to COVID-19-negative patients. Since micronuclei are caused by breakage in chromosomes, the resulting noticeable upsurge of this cytogenetic parameter in COVID-19 patients indicates mutagenicity of buccal epithelial cells, mainly due to genomic instability. Numerous studies have been conducted on exfoliated buccal cells to investigate the cytogenetic changes in tobacco chewers [14], smokers [15], alcoholics [16], painters [17], and those with breast carcinoma [18]. All this research revealed there is a noticeable upsurge in the frequencies of micronuclei occurrences as compared to normal healthy individuals, predicting high risk for the development of oral carcinomas. The quantitative assessment of micronuclei frequency in buccal mucosa serves as a valuable biomarker for oral cancer susceptibility and progression to advanced stages. This had been corroborated by a study comparing the oral mucosa of healthy individuals, those with epithelial dysplasia, and patients with oral squamous cell carcinoma [19].

8-OHdG in buccal cells

Prieto et al., in 2023, showed the 8-OHdG expression in the buccal cells of cancer patients localized in the cytoplasm and nucleus [20]. In this study, the 8-OHdG was expressed in the cytoplasm as granules and around the rim of the nucleus, and based on intensity, it was divided into mild, moderate, and severe. The 8-OHdG is formed due to excessive endo- and exogenous reactive oxygen species via oxygen free radical production as hydroxyl radical (OH+), damaging the DNA strands. The DNA strand with hydroxyl radical affecting the nitrogen base guanosine due to a lower oxidation potential forms C8-hydroguanosine, and by one electron abstraction at the second position leads to the formation of 8-OHdG. This has a promutagenic ability to pair with adenosine instead of cytosine, promoting GC/TA transversion mutations. Thus, 8-OHdG can be used as a biomarker for oxidative stress, aging, and carcinogenesis [21]. This study, for the first time in exfoliated buccal cells, has identified the localization of 8-OHdG in the nuclear rim and cytoplasm as fine to coarse granules. In COVID-19, there was increased expression of 8-OHdG, indicating there was increased oxidative stress in the buccal cells, and can be used as a promising biomarker in assessing the severity of COVID-19.

8-OHdG as a diagnostic marker

There are few studies correlating increased expression of 8-OHdG associated with various cancers, such as oral squamous cell [20], cervical [22], hepatocellular [23], papillary thyroid and prostatic adenocarcinoma [24], colorectal carcinoma [25], lung carcinoma [26], periodontal disease [27], stroke and vascular diseases [28], steatohepatitis [29] and Alzheimer's diseases [30]. Elevated levels of 8-OHdG in oral cancer tissues have been correlated with tumor aggressiveness, metastasis, and poor prognosis. By developing the 8-OHdG localization and assessing its level of expression, it can be used as a screening tool in identifying oral lesion pathologies and early prevention of cancer-related mortality and morbidity. Antioxidant therapies, DNA repair modulators, and anti-inflammatory agents represent potential treatment options for reducing oral epithelial cell damage and improving clinical outcomes in COVID-19 patients.

Mechanism of DNA damage in SARS-CoV-2 infection

**Figure 4:**
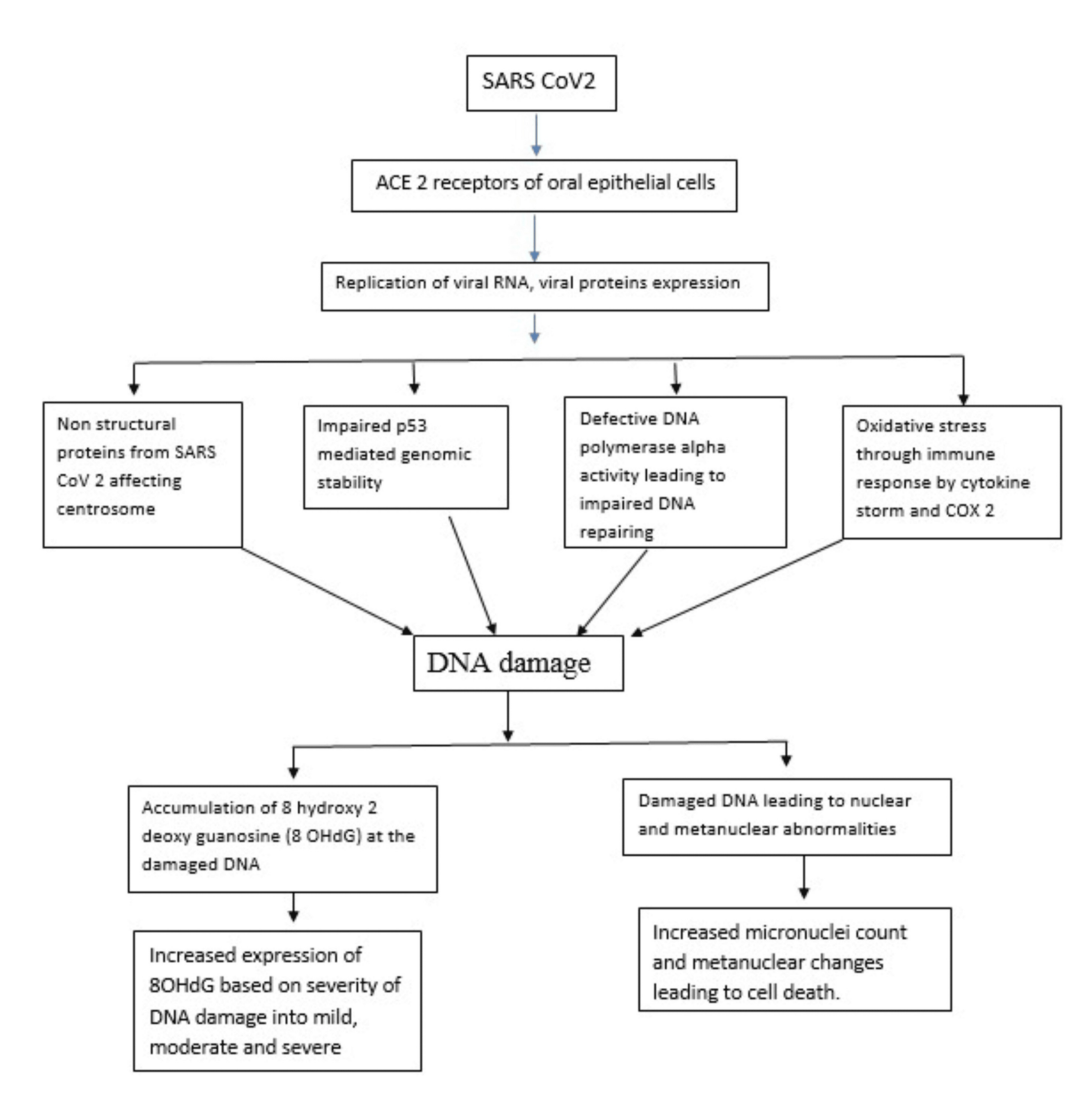
Possible mechanism of DNA damage in SARS-CoV-2 infected buccal cells from this study findings This diagrammatic representation is solely created by corresponding author for our study

SARS-CoV-2 binds to the ACE and TMPRSS2 receptors present on the cell surface and injects its genome into the human nucleus. The SARS-CoV-2 genome comprises of open reading frames (ORF) which are cleaved into nonstructural proteins (nsp) [31]. These nonstructural proteins from SARS-CoV-2 reduce the DNA polymerase alpha, impair the p53 functions, destabilize the centromere, and increase a massive immune response by cytokine storm [32] (Figure 4).

This defective DNA polymerase alpha level leads to impaired DNA repairing, impaired p53 stability leads to genomic instability in human DNA, centrosome instability leads to abnormal genetic material division, and excessive immune response leads to increased oxidative stress [33] and increased accumulation of 8-OHdG [34].

Correlation of 8-OHdG and micronuclei count in COVID-19 patients

In this study, there were coarse granules of 8-OHdG in the cytoplasm and localization in the nucleus, indicating that SARS-CoV-2 produces oxidative DNA damage. There was an increase in micronuclei count and oxidative DNA damage in the COVID-19 patients, indicating that the increased oxidative stress produced by the SARS-CoV-2 virus leads to improper nuclear division and increased nuclear fragments enclosed as micronuclei. The micronuclei, consisting of oxidative DNA accumulation, when localised for 8-OHdG, appear as granulated staining in the cytoplasm. Immunostaining shows coarse staining in cytoplasm and entire nuclear material in severe immunolocalization, indicating that SARS-CoV-2 produces DNA damage and fragments that are dispersed in the cytoplasm, which could be either micronuclei or just simple DNA fragments. These DNA fragments are even dispersed outside the cell into the saliva that appears as artefacts in the smear, and are actually the 8-OHdG localized DNA fragments. There is no literature explaining this kind of staining pattern in the buccal cells, and this is the first study to show this.

The levels of 8-OHdG have been assessed in various studies, such as non-insulin dependent diabetes mellitus [35], cardiovascular diseases [36], atherosclerotic plaque formation in stroke, Alzheimer's disease [37], chronic kidney diseases [38], and Huntington's disease [39]. In all these conditions, the levels of 8-OHdG have been elevated, and the levels vary not only in pathogenesis but also, the disease outcomes and prognosis were related to levels of 8-OHdG. However, in the case of breast cancer, the low levels of 8-OHdG are associated with poor prognosis, and the reason is probably due to the rich antioxidant mechanism within the breast tissue [24] 

Genotoxic assessment by epithelial to inflammatory cell ratio

In this study, the epithelial to inflammatory cell count in COVID-19 patients was 14.03, which was significantly lower compared to COVID-19 negative patients. The number of inflammatory cells was very high in COVID-19-positive patients, specifically, the inflammatory cell population of neutrophils and lymphocytes. The antimicrobial functions of inflammatory cells include adhesion of microbes, production of reactive oxygen species, and damage to membranes and genetic material [40]. Neutrophils contribute to cancer progression by suppressing the cytotoxic and helper cells and stimulate the tumor angiogenesis and metastasis by creating a niche in the epithelial barrier [41]. This ratio can be useful to correlate the severity of immune response to the SARS-CoV-2 infection with its short-term genotoxic damage and its impact, with the severity of COVID-19. Even though this study did not follow up to find the severity of COVID-19, this can be helpful in the future. The lymphocyte count was more comparable with the neutrophil count as a response to infection, but there is no literature explaining the importance of this and its role in saliva.

Limitations of the study

This is a cross-sectional study, providing the genotoxic effects at a single point in time. Without longitudinal data, it is difficult to determine whether the observed DNA damage in COVID-19 patients is transient (e.g., a result of the acute infection) or whether it persists long-term, potentially contributing to later cancer risk or other complications. While the study mentions mechanisms like DNA polymerase alpha dysfunction and p53 impairment, there is no detailed exploration of the specific molecular pathways through which SARS-CoV-2 induces DNA damage. Further investigation into how the virus disrupts these cellular processes at the molecular level could provide deeper insights into potential therapeutic targets. This study compares COVID-19-positive and -negative individuals, but it does not provide comparative data from other high-risk groups, such as smokers, alcohol users, or individuals with known oral pre-cancerous lesions. This would help to contextualize whether the genotoxic effects seen in COVID-19 patients are unique or similar to those seen in other populations known to have higher risks for oral cancer.

## Conclusions

This study concludes that SARS-CoV-2 has a genotoxic effect by increasing the micronuclei count and creates oxidative DNA damage by increasing the 8-OHdG expression in the exfoliated buccal cells. This confirms that the oxidative DNA damage has a direct impact on the formation of micronuclei. Also, there is a decreased epithelial-to-lymphocyte ratio in COVID-19-positive patients' buccal smears, indicating higher inflammatory cells in the smear. This causes oxidative DNA damage by the release of reactive oxygen species, with the genotoxic effect leading to more micronucleated cells. The micronuclei count and 8-OHdG expression levels can be used as an early biomarker to identify oral lesions, and the epithelial to inflammatory cell ratio can be used in assessing the severity of COVID-19 infection.
